# Charge Transport in UV-Oxidized Graphene and Its Dependence on the Extent of Oxidation

**DOI:** 10.3390/nano12162845

**Published:** 2022-08-18

**Authors:** Hwa Yong Lee, Mohd Musaib Haidari, Eun Hee Kee, Jin Sik Choi, Bae Ho Park, Eleanor E. B. Campbell, Sung Ho Jhang

**Affiliations:** 1 School of Physics, Konkuk University, Seoul 05029, Korea; 2 EaStCHEM, School of Chemistry, Edinburgh University, David Brewster Road, Edinburgh EH9 3FJ, UK

**Keywords:** graphene oxide, defect density, transport gap, band gap, metal–insulator transition, 2D Mott VRH

## Abstract

Graphene oxides with different degrees of oxidation are prepared by controlling UV irradiation on graphene, and the charge transport and the evolution of the transport gap are investigated according to the extent of oxidation. With increasing oxygenous defect density $n_{D}$, a transition from ballistic to diffusive conduction occurs at $n_{D}≃10^{12}$ cm${}^{-2}$ and the transport gap grows in proportion to $\sqrt{n_{D}}$. Considering the potential fluctuation related to the e−h puddle, the bandgap of graphene oxide is deduced to be $E_{g}≃30\sqrt{n_{D}\;\left(10^{12}\;{\mathrm{cm}}^{-2}\right)}$ meV. The temperature dependence of conductivity showed metal–insulator transitions at $n_{D}≃0.3\times 10^{12}$ cm${}^{-2}$, consistent with Ioffe–Regel criterion. For graphene oxides at $n_{D}\geq 4.9\times 10^{12}$ cm${}^{-2}$, analysis indicated charge transport occurred via 2D variable range hopping conduction between localized $sp^{2}$ domain. Our work elucidates the transport mechanism at different extents of oxidation and supports the possibility of adjusting the bandgap with oxygen content.

## 1. Introduction

Graphene is a two-dimensional semimetal with high conductivity and mobility [1,2], and is a promising candidate for applications within electronics and optoelectronics. However, a bandgap $E_{g}$ is required for certain applications and various methods have been attempted to controllably induce a bandgap in graphene. While a confinement-induced bandgap is widely investigated in graphene nanoribbons [3], a bandgap can also be induced by breaking the symmetry of the graphene lattice. As an approach to break the symmetry, functionalization of graphene with foreign atoms [4,5,6] such as oxygen, fluorine, and hydrogen has been tested to open a finite bandgap. For graphene oxide (GO), the bandgap is suggested to be tuned by the extent of oxidation, and $E_{g}≃$ 2.6–6.5 eV is theoretically expected for fully oxidized graphene (O/C = 50%) [7,8,9,10].

The most common method to produce GO is based on wet chemistry, consisting of oxidation of graphite in strong acids, followed by a liquid exfoliation [4,11]. The degree of oxidation can be tuned by subsequent reduction via thermal or chemical treatment [12], and the transition from insulator to semimetal with increasing reduction of GO has been reported [13,14]. Despite the advantage for large-scale production of GO, this production method introduces contamination, and alternative dry oxidation methods such as plasma [4,15] and UV treatments [16,17] have been developed. Plasma oxidation produces GO by exposing graphene to an oxygen plasma, and the semimetallic graphene undergoes a transition into an insulator according to the time of exposure to the plasma [9,18]. UV/ozone treatment also controls the degree of oxidation of graphene with UV exposure time, and both methods provide convenient control over the extent of oxidation. In addition, UV oxidation causes less distortion of the graphene lattice [19,20] compared to the energetic plasma collisions that can introduce topological defects.

Transport studies of GO and observation of metal–insulator transition via oxidation or reduction reactions have been reported [9,13,14,18], but a systematic study of the transport mechanism and evolution of the transport gap for different degrees of oxidation is still required. In this paper, we produced GO samples with different extents of oxidation through UV treatment and systematically investigated the transport mechanism and the evolution of the transport gap across the metal–insulator transition.

## 2. Experimental

**Figure 1:**
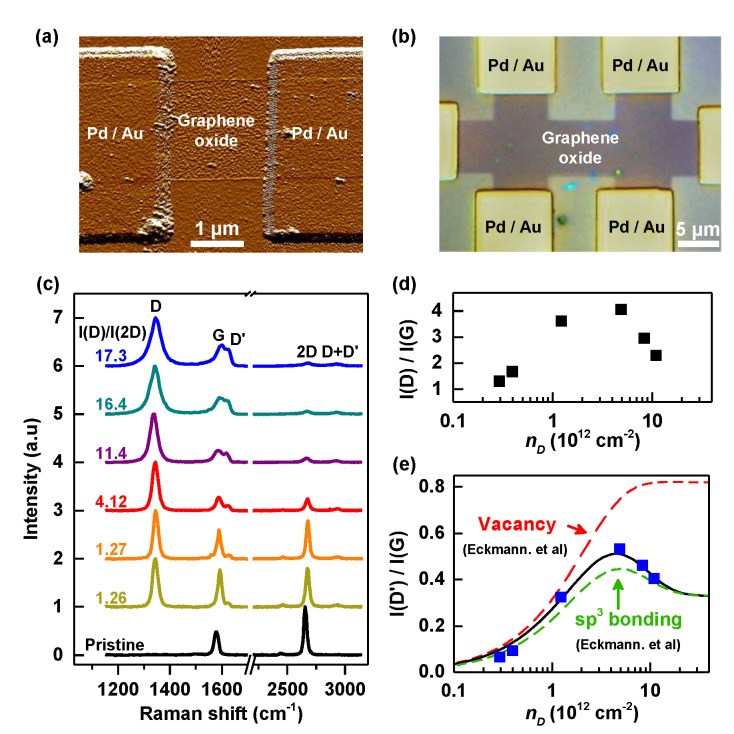
(**a**) Atomic force microscope image of a typical graphene FET, irradiated with UV for oxidation. (**b**) Optical image of a CVD-grown graphene device with Hall-bar geometry. (**c**) The evolution of Raman spectra of graphene devices exposed to UV light arranged according to the value of I(D)/I(2D). (**d**) Values of $n_{D}$ deduced from I(D)/I(G) for six graphene devices irradiated with UV (**e**) $I\left(D^{\prime }\right)/I\left(G\right)$ as a function of $n_{D}$. The black solid line is a fit to Equation (2). Dashed lines are the evolution of $I\left(D^{\prime }\right)/I\left(G\right)$ either for vacancies or $sp^{3}$ sites, suggested from Ref. [21].

Our experiments were conducted on seven graphene field-effect-transistors (FETs) fabricated using exfoliated graphene on SiO${}_{2}$ (300 nm)/Si substrates, and a device with Hall-bar geometry using graphene grown by chemical vapor deposition. For all devices, Pd (20 nm)/Au (20 nm) electrodes were deposited by e-beam lithography. Typical images of the devices are shown in Figure 1a,b. For those seven FETs prepared using exfoliated graphene, the size of the channel was unified to 2 μm × 2 μm (width × length) via oxygen plasma etching to compare later the characteristics depending on the extent of the graphene oxidation. The graphene devices were then oxidized by irradiating UV with a wavelength of 172 nm at an intensity of 20 mW/cm${}^{2}$ under ambient conditions (humidity with 30 to 40%). The extent of oxidation was roughly controlled by the irradiation time. Among seven graphene FETs, groups of two devices were exposed to UV light for 15, 20, and 25 s, respectively, leaving one pristine graphene FET for reference. A laser with excitation energy of 2.33 eV was used to obtain Raman spectra of the oxidized graphene. Charge transport characteristics of the seven FETs were studied using a vacuum probe station and a Keithley 4200 semiconductor characterization system in Core Facility Center for Quantum Characterization/Analysis of Two-Dimensional Materials & Heterostructures for the temperature 77 < *T* < 400 K. The graphene device with Hall-bar geometry was investigated after UV oxidation by using a quantum design PPMS for lower *T* down to 2 K and magnetic fields up to 7 tesla.

## 3. Result and Discussion

Figure 1c presents Raman spectra of pristine graphene and six graphene FETs exposed to UV light for 15, 20, or 25 s. Defect-activated *D* (∼1345 cm${}^{-1}$), $D^{\prime }$ (∼1625 cm${}^{-1}$), and $D+D^{\prime }$ (∼2930 cm${}^{-1}$) peaks appeared for the graphene samples irradiated with UV, in addition to *G* (∼1580 cm${}^{-1}$) and 2D (∼2650 cm${}^{-1}$) peaks of pristine graphene [22]. The two graphene FETs irradiated for the same time resulted in rather different Raman spectra, possibly due to the different degrees of PMMA residues remaining on the graphene samples. Hence, in Figure 1c, Raman spectra were arranged with respect to the ratio between the *D* and the 2D peak intensities, I(D)/I(2D), as the rise of *D* and the suppression of 2D peaks with increasing defect density were reported extensively as a means of quantifying defective graphene including graphene oxide [21,23,24,25]. Previous works on defective graphene introduced the local activation model to explain the evolution of Raman spectra, and in this model the ratio between the *D* and *G* peak intensities, I(D)/I(G), allows us to estimate the defect density $n_{D}$, which corresponds to the degree of oxidation in our experiments [21],

(1) $$\frac{I(D)}{I(G)}\;=\;C_{A,D}\;\frac{r_{A,D}^{2}-r_{S,D}^{2}}{r_{A,D}^{2}-2r_{S,D}^{2}}\;\left[e^{-\pi^{2}r_{S,D}^{2}\;n_{D}}-e^{-\pi^{2}\left(r_{A,D}^{2}-r_{S,D}^{2}\right)\;\;n_{D}}\right]$$

Here, $C_{A,D}$ is a parameter related to the electron–phonon coupling of the *D* peak phonon, and $r_{S,D}$ and $r_{A,D}$ are values that indicate the size of the defect site. To deduce $n_{D}$, we assumed $C_{A,D}=6$, which agrees well with the excitation laser of 532 nm and the maximum value of I(D)/I(G)≃ 4 observed in our experiment [26,27] with $r_{S,D}=1$ nm and $r_{A,D}=3$ nm [21]. As shown in Figure 1d, values of $n_{D}$ are deduced to be between 0.29 and 11 (×10 ${}^{12}$ cm ${}^{-2}$) for the graphene samples irradiated with UV light.

To discuss the nature of the defects, we now inspect the ratio between the $D^{\prime }$ and the *G* peak intensities, $I\left(D^{\prime }\right)/I\left(G\right)$, which is sensitive to the nature of the defect. Figure 1e shows $I\left(D^{\prime }\right)/I\left(G\right)$ as a function of $n_{D}$ estimated for the six irradiated graphene samples. For low defect densities, $I\left(D^{\prime }\right)/I\left(G\right)$ increases with $n_{D}$, and then starts to decrease for $n_{D}\geq 4.9\times 10^{12}$ cm${}^{-2}$ presenting a maximum value of ≃ 0.5. The ratio between the $D^{\prime }$ and the *G* peak intensities can be fitted with the following Equation (2).

(2) $$\begin{array}{cc} \frac{I(D^{\prime })}{I(G)}\;=\; & C_{A,D^{\prime }}\;\frac{r_{A,D^{\prime }}^{2}-r_{S,D^{\prime }}^{2}}{r_{A,D^{\prime }}^{2}-2r_{S,D^{\prime }}^{2}}\;\left[e^{-\pi^{2}r_{S,D^{\prime }}^{2}\;n_{D}}-e^{-\pi^{2}\left(r_{A,D^{\prime }}^{2}-r_{S,D^{\prime }}^{2}\right)\;n_{D}}\right]\\ & +C_{S}\;\left(1-e^{-\pi^{2}r_{S,D^{\prime }}^{2}\;n_{D}}\right) \end{array}$$

Here, $C_{S}$ is a parameter related to the defect type and $C_{A,D^{\prime }}$ is a parameter related to the electron–phonon coupling of the $D^{\prime }$ peak phonon, with $r_{A,D^{\prime }}$ and $r_{S,D^{\prime }}$ being the length scales of the defect sites. Our data are best fitted with Equation (2) (solid line) when $C_{S}$ = 0.33 and $C_{A,D^{\prime }}$ = 0.63 (Figure 1e). Eckmann et al. [21] derived from their experiments $C_{S}$ = 0.33 for $sp^{3}$ sites and $C_{S}$ = 0.82 for vacancies. Excellent agreement with $C_{S}$ = 0.33 suggests the oxidation of our graphene through UV treatment, forms $sp^{3}$ bonds. Dashed lines in Figure 1e show the evolution of $I\left(D^{\prime }\right)/I\left(G\right)$ for graphene with either vacancies or $sp^{3}$ sites, calculated with $C_{A,D^{\prime }}$ = 0.5, $r_{S,D^{\prime }}=1.4$ nm and $r_{A,D^{\prime }}=2.6$ nm from ref. [21]. Note the slight mismatch with the curve for $sp^{3}$ sites is due to the different value of $C_{A,D^{\prime }}$ determined for our samples.

**Figure 2:**
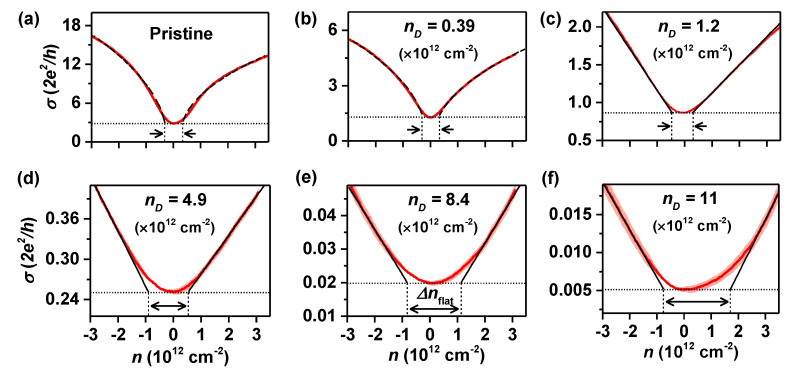
Transfer curves of oxidized graphene FETs with different $n_{D}$. The red shade shows the standard deviation of repeated measurements. (**a**) pristine graphene; (**b**) $n_{D}=0.39\times 10^{12}$ cm${}^{-2}$; (**c**) $n_{D}=1.2\times 10^{12}$ cm${}^{-2}$; (**d**) $n_{D}=4.9\times 10^{12}$ cm${}^{-2}$; (**e**) $n_{D}=8.4\times 10^{12}$ cm${}^{-2}$; (**f**) $n_{D}=11\times 10^{12}$ cm${}^{-2}$. Black dotted lines show the minimum conductivity $\sigma_{\min}$. Dashed lines in (**a**,**b**) are fits to $\sigma \propto \sqrt{n}$, and solid lines in (**c**–**f**) are linear fits to the transfer curves. With increasing $n_{D}$, transfer curve becomes more flattened near n=0, as indicated by the width $\Delta n_{\mathrm{flat}}$.

Regarding $n_{D}$ as an oxygenous defect density and a measure of the degree of oxidation of graphene, we investigate the charge transport characteristics depending on $n_{D}$ in oxidized graphene devices. Figure 2 displays transfer characteristics of the oxidized graphene FETs at different $n_{D}$, measured with a fixed drain-source bias $V_{\mathrm{DS}}$ of 1 mV. A back-gate voltage $V_{g}$ is applied over the 300 nm thick SiO${}_{2}$, and the carrier density *n* is given by $n=\alpha (V_{g}-V_{\mathrm{CNP}})$ with α = 7.2 × 10${}^{10}$ cm${}^{-2}$ using the parallel capacitor model [28]. Here, $V_{\mathrm{CNP}}$ is the voltage at the charge-neutrality point. The conductivity σ monotonically decreases with increasing $n_{D}$. Compared to the pristine graphene, the conductivity of oxidized graphene is, for example, ∼1000 times smaller at $n_{D}=11\times 10^{12}$ cm${}^{-2}$. In addition, the conductivity becomes flattened near n=0 with increasing $n_{D}$, discussed in terms of the so-called “transport gap” in the next paragraph. We also note for samples with $n_{D}<10^{12}$ cm${}^{-2}$ it follows $\sigma \propto \sqrt{n}$ (Figure 2a,b), while for $n_{D}>10^{12}$ cm${}^{-2}$ it shows σ∝n outside the flat area (Figure 2c–f). This behavior implies that charge transport transitioned from ballistic to diffusive transport at the oxidation density of $n_{D}≃10^{12}$ cm${}^{-2}$. It is known that $\sigma \propto \sqrt{n}$ in ballistic graphene [28,29] and σ∝n in diffusive graphene [30,31]. The estimation of the mean free path $l=\frac{\sigma h}{2e^{2}}\;\cdot \;\frac{1}{\sqrt{\pi n}}$[28,32] gives l≃ 4 μm for our pristine graphene and l≃ 1.5 μm for the GO FET with $n_{D}=0.39\times 10^{12}$ cm${}^{-2}$, which are twice as large and comparable to the channel length (2 μm), respectively. Additionally, l≃600,130, and 5 nm, estimated for GO FETs with $n_{D}$ = 1.2, 4.9, and 11 (×10${}^{12}$ cm${}^{-2}$), respectively, meet the condition for diffusive transport (*l* < channel length), consistent with our observation.

**Figure 3:**
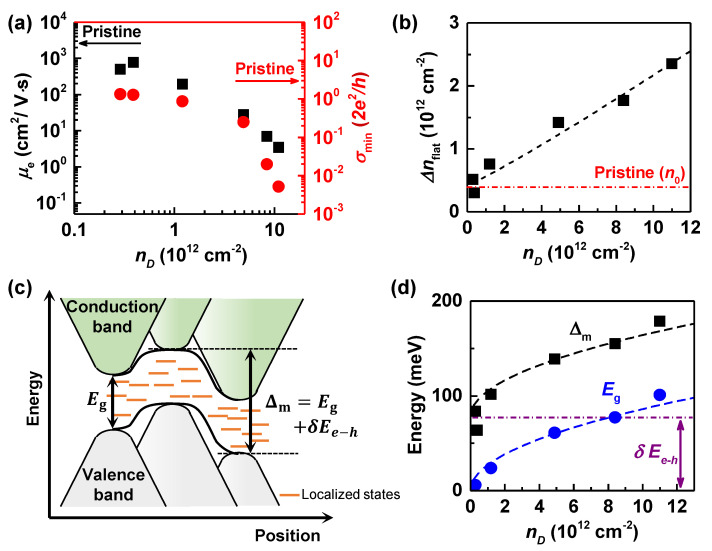
(**a**) Electron side mobility $\mu_{e}$ (black square) and conductivity minimum $\sigma_{\min}$ (red circle) of graphene oxide as a function of $n_{D}$. Arrows indicate the values of $\mu_{e}$ and $\sigma_{\min}$ for pristine graphene. (**b**) $\Delta n_{\mathrm{flat}}$ versus $n_{D}$. The black dashed line is a linear fit to the data. Note y-intercept $n_{0}$, associated with the electron–hole puddle in pristine graphene. (**c**) Schematic band diagram of graphene oxide and the illustration of an electron-hole puddle, affecting the transport gap observed. (**d**) Transport gap (black square) and inferred bandgap (blue circle) of graphene oxide as a function of $n_{D}$. Dashed lines are fitting curves with $\sqrt{n_{D}}$ and the dot-dash line indicates the size of potential fluctuation due to e−h puddle.

The change in the transport mechanism observed in GO FETs happens because the scattering with oxygenous defects becomes more frequent as the extent of the oxidation increases. Figure 3a shows how the conductivity minimum $\sigma_{\min}$ and electron side mobility $\mu_{e}$ are reduced with increasing extent of oxidation. Field-effect mobility estimated from the electron side ($\mu_{e}=\frac{1}{e}\;\cdot \;\frac{d\sigma }{dn}$ [24]), decreases from $\mu_{e}∼$ 2900 (pristine) to ∼ 500 ($n_{D}=0.29\times 10^{12}$ cm${}^{-2}$) and ∼ 3.6 cm${}^{2}$/V·s ($n_{D}=11\times 10^{12}$ cm${}^{-2}$) with increasing $n_{D}$. These results are also consistent with μ≃30−2 cm${}^{2}$/V·s, reported for reduced graphene oxides at $n_{D}≃5-11\;(\times$10${}^{12})$ cm${}^{-2}$, synthesized by using a modified Hummer’s method [26,33]. On the other hand, with the oxidation of graphene, the bandgap opens and disorder-induced localized states appear inside the bandgap, resulting in a transport gap $\Delta_{m}$ related to $\Delta n_{\mathrm{flat}}$ in Figure 2[34,35]. To discuss the dependence of the transport gap on the oxygen content, we display in Figure 3b the flattened width $\Delta n_{\mathrm{flat}}$, observed in Figure 2, as a function of $n_{D}$. $\Delta n_{\mathrm{flat}}$ gradually increases with $n_{D}$, while $\Delta n_{\mathrm{flat}}=0.42\times 10^{12}$ cm${}^{-2}$ for pristine graphene, associated with residual carrier densities $n_{0}$ originating from the electron(*e*)–hole(*h*) puddle [36]. The transport gap, estimated from $\Delta_{m}=\hbar v_{F}\sqrt{\pi \Delta n_{\mathrm{flat}}}$ [37,38,39], overestimates the actual bandgap due to the existence of disorder potentials near the charge neutrality point (e−h puddle), as illustrated in Figure 3c. Taking into account the potential fluctuation related to the e−h puddle, $\delta E_{e-h}=\hbar v_{F}\sqrt{\pi \Delta n_{0}}≃$ 78 meV from the pristine graphene device, we can infer the bandgap of UV-oxidized graphene at the different oxygenous defect densities. With $E_{g}≃\Delta_{m}-\delta E_{e-h}$, the inferred bandgap is presented as a function of $n_{D}$ in Figure 3d, together with the transport gap observed, $\Delta_{m}$. $E_{g}$ is seen to increase in proportion to $\sqrt{n_{D}}$, according to the relationship $E_{g}≃30\sqrt{n_{D}\;\left(10^{12}\;{\mathrm{cm}}^{-2}\right)}$ meV. Substitution of $n_{D}≃1.9\times 10^{15}$ cm${}^{-2}$ for fully oxidized graphene C${}_{2}$O (epoxide) results in $E_{g}≃1.3$ eV, comparable to $E_{g}≃2.6$–6.5 eV calculated from theory for fully oxidized graphene [7,8,9,10]. Our results support the possibility of continuously adjusting the bandgap by tuning the oxygen content.

**Figure 4:**
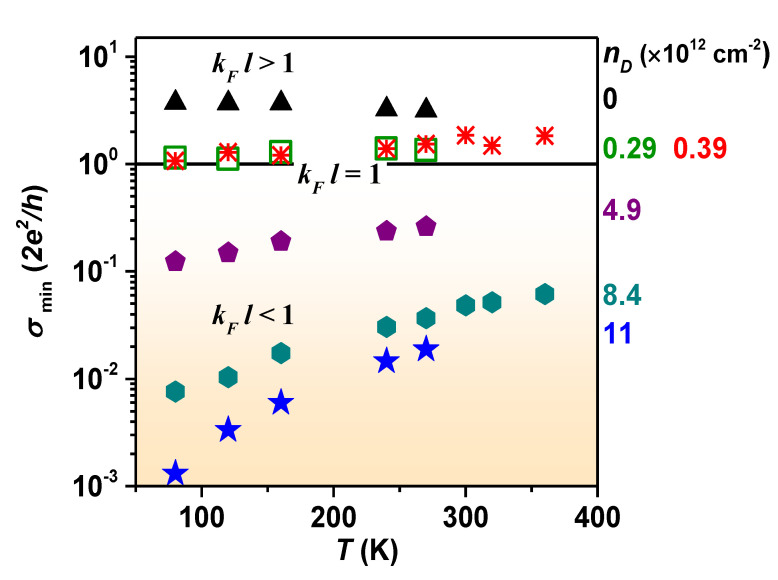
Temperature dependence of conductivity minimum for graphene oxides with different $n_{D}$. Solid line indicates $k_{F}l=1$.

Figure 4 shows the temperature *T* dependence of $\sigma_{\min}$ for graphene oxide with different values of $n_{D}$, studied for 77 < *T* < 400 K. Pristine graphene exhibits metallic behavior in which $\sigma_{\min}$ decreases with *T*. For GO samples with $n_{D}=0.29$ and 0.39 ($\times 10^{12}$ cm${}^{-2}$), conductivity minimum slightly increases with *T*, and the insulating behavior develops further with increasing $n_{D}$. The metal–insulator transition appears for $k_{F}l=1$ and meets the Ioffe–Regel criterion, where $k_{F}$ is the Fermi wavenumber and *l* is the mean free path with $k_{F}l=\sigma_{\min}\;\frac{h}{2e^{2}}$[40]. Considering $E_{g}≃60$ meV for GO at $n_{D}≃5\times 10^{12}$ cm${}^{-2}$, inferred from Figure 3d, the pronounced insulating behavior observed for $n_{D}\geq 4.9\times 10^{12}$ cm${}^{-2}$ reflects that the size of opened bandgap becomes larger than the thermal energy (∼26 meV at room temperature).

**Figure 5:**
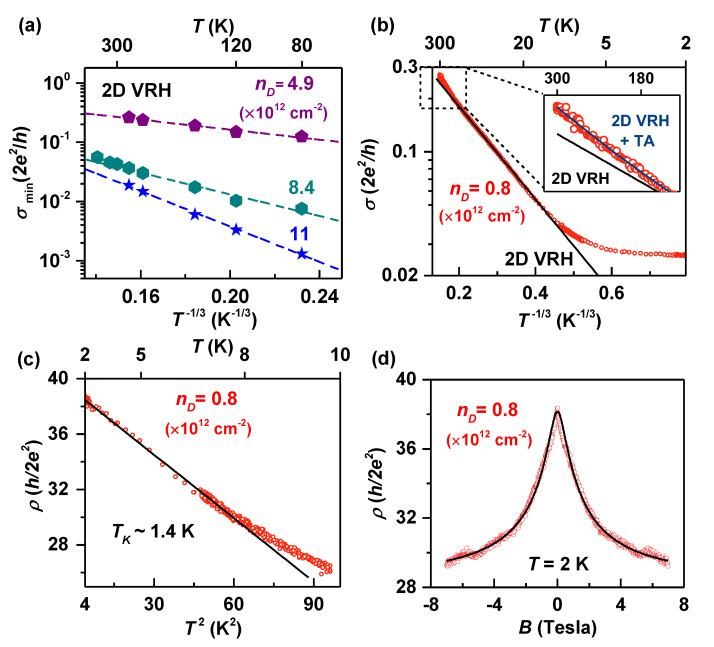
(**a**) $\log\sigma_{\min}$ vs. $T^{-1/3}$ for graphene oxides with $n_{D}$ = 4.9, 8.4, and 11 × 10${}^{12}$ cm${}^{-2}$. (**b**) logσ vs. $T^{-1/3}$ for graphene oxide with $n_{D}$ = 0.8 × 10${}^{12}$ cm${}^{-2}$, measured between 2 < *T* < 300 K. For 15 < *T* < 145 K, logσ is linearly proportional to $T^{-1/3}$ and agrees well with 2D VRH model. (Inset) logσ vs. $T^{-1/3}$, zoomed in for 300 < *T* < 140 K. The data are best fitted considering both 2D VRH and thermal activation conduction. (**c**) ρ vs. $T^{2}$ in the low *T* regime for 2 < *T* < 10 K for GO with $n_{D}=0.8\times$ 10${}^{12}$ cm${}^{-2}$. (**d**) Magnetoresistance of the graphene oxide with $n_{D}=0.8\times 10^{12}$ cm${}^{-2}$, measured at T= 2 K.

In Figure 5a, analysis shows the charge transport in GO samples for larger $n_{D}=4.9,\;8.4$, and $11\times 10^{12}$ cm${}^{-2}$ is localized and the *T*-dependence of $\sigma_{\min}$ is well explained by 2D Mott variable range hopping conduction (2D VRH) [14,41], following $\sigma =\sigma_{0}\exp{\left(\frac{T_{0}}{T}\right)}^{\frac{1}{3}}$. Note that $\log\sigma_{\min}$ is linear with $T^{-1/3}$ in Figure 5a. Characteristic temperature $T_{0}=$ 900, 17,000, and 41,000 K are obtained from the linear fits for $n_{D}=$ 4.9, 8.4, and 11$\times 10^{12}$ cm${}^{-2}$, respectively. The localization length $\xi =\sqrt{\frac{13.8}{k_{B}\mathrm{DOS}(E)T_{0}}}$, estimated for $0<n<3\times 10^{12}$ cm${}^{-2}$, decreases from 32–46 nm ($n_{D}=4.9$) and 9–17 nm ($n_{D}=8.4$) to 6–11 nm ($n_{D}=11\times 10^{12}$ cm${}^{-2}$) with increasing $n_{D}$. Here, $k_{B}$ is the Boltzmann constant, and DOS(*E*) is the density of states of graphene [32]. The reduction of ξ with $n_{D}$ implies the size of the $sp^{2}$ domain decreases as the extent of oxidation increases [14,41]. On the other hand, charge transport in GO samples with smaller $n_{D}=0.23$ and $0.39\times 10^{12}$ cm${}^{-2}$ is not explained by 2D VRH or a thermal activation model. To investigate the charge transport at metal–insulator boundaries in more detail, we prepared an additional GO FET with $n_{D}=0.8\times 10^{12}$ cm${}^{-2}$ in a Hall-bar geometry shown in Figure 1b and studied the four-probe conductivity between 2 < *T* < 300 K. Figure 5b plots logσ of GO with $n_{D}=0.8\times 10^{12}$ cm${}^{-2}$ as a function of $T^{-1/3}$. Whereas for 15 < *T* < 145 K, logσ is linearly proportional to $T^{-1/3}$ and agrees well with the 2D VRH model, σ is a little larger than the linear fitting curve for 145 < *T* < 300 K as shown in the inset of Figure 5b. Additionally, σ becomes saturated for 2 < *T* < 15 K, deviating from the linear dependence.

In the temperature range of 145 < *T* < 300 K, the *T*-dependence of the conductivity can be explained by considering 2D VRH and thermal activation conduction (TA) together. The data are fitted well with $\sigma =\sigma_{0}\exp{\left(\frac{T_{0}}{T}\right)}^{\frac{1}{3}}+\sigma_{1}\exp\left(\frac{E_{a}}{k_{B}T}\right)$, as seen from the inset of Figure 5b, and an activation energy of $E_{a}≃47$ meV is obtained. This value is larger than $E_{g}≃27$ meV inferred from Figure 3d and can be associated with the influence of the e−h puddle ($\delta E_{e-h}≃$ 78 meV). The saturation of σ below T<15 K is analyzed by considering the Kondo effect and presented in Figure 5c. The Kondo effect occurs when the charge carriers interact with the local magnetic moment of defects and the *T*-dependence of electrical resistivity ρ is given as follows in the low-temperature regime [42].

(3) $$\rho =\rho_{c}+\rho_{K}\left[1-{\left(\frac{\pi }{2}\right)}^{4}{\left(\frac{T}{T_{K}}\right)}^{2}\right]$$

Here, $\rho_{K}$ is the Kondo resistivity at 0 K, and $\rho_{c}$ is the temperature-independent resistivity parameter. $T_{K}$ is the Kondo temperature, and the stronger the coupling between the magnetic moment and the charge carrier, the greater the value of $T_{K}$[42]. In Figure 5c, ρ is plotted versus $T^{2}$, and $\rho_{K}≃20\frac{h}{e^{2}}$, $\rho_{c}≃0.049\frac{h}{e^{2}}$ and $T_{K}$ = 1.4 K are deduced from a linear fit. The obtained value of $T_{K}$ = 1.4 K suggests rather weak coupling between the magnetic moment of defects and charge carriers in UV-oxidized graphene with $n_{D}=0.8\times 10^{12}$ cm${}^{-2}$.

Figure 5d shows the magnetoresistance (MR) of the graphene oxide at $n_{D}=0.8\times 10^{12}$ cm${}^{-2}$, measured at T= 2 K. A negative MR was observed in which ρ decreased as the magnetic field *B* increased up to 7 teslas. The negative MR is analyzed with the following equation [43] including both strong and weak localization effects.

(4) $$\frac{\rho (B)-\rho (0)}{\rho (0)}=\frac{l_{\phi }}{l_{c}}\left[\frac{\Psi \left(\frac{2B_{c}+B_{\phi }+B_{e}}{B}+\frac{1}{2}\right)-\Psi \left(\frac{B_{c}+B_{\phi }}{B}+\frac{1}{2}\right)}{\ln\left(1+\frac{B_{e}}{B_{c}+B_{\phi }}\right)}-1\right]$$

Here, Ψ(x) is the digamma function, and the characteristic length $l_{i}$ is related to the characteristic magnetic field $\left(B_{\left(i=c,\phi ,e\right)}=\frac{\hbar }{4e{l_{i}}^{2}}\right)$. A localization length of $l_{c}≃$ 160 nm, phase coherence length of $l_{\phi }≃46$ nm, and elastic scattering length of $l_{e}≃$ 13 nm are obtained from the fitting of Equation (4) to the MR data. $l_{\phi }$ is three times smaller than $l_{c}$, implying a dominant role of weak localization in the negative MR for the GO with $n_{D}=0.8\times 10^{12}$ cm${}^{-2}$.

## 4. Conclusions

In summary, we have prepared graphene-oxide FETs with different degrees of oxidation by controlling the UV irradiation time on graphene, and investigated the charge transport and the evolution of the transport gap according to the extent of oxidation. With increasing oxygenous defect density $n_{D}$, the charge transport transitioned from ballistic to diffusive conduction around $n_{D}≃10^{12}$ cm${}^{-2}$ and the transport gap grew in proportion to $\sqrt{n_{D}}$. Taking into account the potential fluctuation related to the e−h puddle, we suggested the bandgap of GO to be $E_{g}≃30\sqrt{n_{D}\;\left(10^{12}\;{\mathrm{cm}}^{-2}\right)}$ meV. The temperature dependence of the conductivity showed metal–insulator transitions at $n_{D}≃0.3\times 10^{12}$ cm${}^{-2}$ at the point where $k_{F}l≃1$, which meets the Ioffe–Regel criterion. For GO with $n_{D}\geq 4.9\times 10^{12}$ cm${}^{-2}$, analysis indicated charge transport occurred via 2D variable range hopping conduction between localized $sp^{2}$ domains with the localization length decreasing with $n_{D}$. Finally, the Kondo effect and negative MR in the low-temperature regime were studied in GO with $n_{D}=0.8\times 10^{12}$ cm${}^{-2}$. 

## Data Availability

Not applicable.
